# Socioeconomic Position and Excessive Daytime Sleepiness: A Systematic Review of Social Epidemiological Studies

**DOI:** 10.3390/clockssleep4020022

**Published:** 2022-05-16

**Authors:** Imene Bendaoud, Faustin Armel Etindele Sosso

**Affiliations:** 1 Clinical Pharmacology, Faculty of Medicine, University of Montreal, Montreal, QC H3T 1J4, Canada; 2 Global Health and Ecoepidemiology, Redavis Institute, Montreal, QC H3S 1X5, Canada; faustin.armel.etindele.sosso@umontreal.ca

**Keywords:** sleepiness, systematic review, social class, health status disparities, economics, global health

## Abstract

The objectives of this empirical study are to describe and discuss the current literature available on the relationship between excessive daytime sleepiness (EDS) and the socioeconomic position (SEP) as well as to provide recommendations for consideration of SEP in sleep medicine and biomedical research. Databases Medline/PubMed, Web of Science, Google scholar and Scopus were screened from January 1990 to December 2020 using PRISMA guidelines and 20 articles were included in the final synthesis. Nineteen studies were cross-sectional and one study was longitudinal. Among these studies, 25.00% (n = 5) are focused on children and adolescent and the remaining 75.00% (n = 15) focused on adults and seniors. Ages ranged from 8 to 18 years old for children/adolescent and ranged from 18 to 102 years old for adults. Main SEP measures presented in these studies were education, income, perceived socioeconomic status and employment. The sample size in these studies varied from N = 90 participants to N = 33,865 participants. Overall, a lower educational level, a lower income and full-time employment were associated with EDS. Symptoms of EDS are prevalent in women, especially those with a low income or no job; and children and adolescents with difficult living conditions or working part time reported more sleep disturbances. SEP is already considered as an important determinant for many health outcomes, but if SEP is embedded in the experimental design in psychosomatic research, biomedical research and clinical practice as a constant variable regardless of outcome; it will move forward future investigations.

## 1. Introduction

Sleep is an essential state to restore and revitalize physiological and mental processes, occupying up to a third of a person’s lifetime. The prevalence of sleep disturbances has been increasing steadily over the past decades [1,2,3,4,5], alongside modern societal and technological changes that influence quality and quantity of sleep. 

Alterations in sleep have been linked to a wide range of health issues including cardiovascular or metabolic disorders [6,7,8,9,10,11,12], as well as an increase in workplace and occupational health issues due to sleep disturbances such as excessive daytime sleepiness (EDS) [13,14,15]. According to the National Sleep Foundation 2000 Omnibus Sleep in America Poll: 43% of adults report that they are so sleepy during the day to the point that it interferes with their daily activities a few days per month or more [16]. 

Sleepiness is an imperative biological function defined by the likelihood of falling asleep, however, when this tendency is increased and the propensity to sleep becomes compulsive, it is referred to as excessive daytime sleepiness (EDS) [17,18]. The latter is defined variously as daytime somnolence or when wakefulness is difficult to maintain [17,18]. This burdensome disorder has been widely studied in order to understand its physiological and neurological substrates; however, few studies have looked into the association between EDS and socioeconomic indicators of social class or socioeconomic position (SEP) [7,13,19,20,21,22,23]. 

It has been previously shown that a lower socioeconomic status, including employment, educational level or income, is an important stressor and impacts sleep negatively [24]. Despite the known adverse consequences of excessive sleepiness during the day, literature is scarce on the topic. 

Substantial evidence has shed some light on the graded association of SEP and health problems [8,19,25,26,27,28,29,30]. However, despite the numerous findings linking SEP with health, there is an enormous lack of research conducted on the relationship between SEP and sleep disturbances. Due to the high implication of SEP in health status and the public health issue that sleep disturbances represent [31], a better understanding of this relationship is necessary. 

A systematic review seemed more appropriate to investigate this question, because the indicators needed to assess SEP, such as income, education or employment, are used differently depending on the context. Therefore, the heterogeneity of the results prevents a statistical combination of the findings necessary for a meta-analysis. The present systematic review was performed for various purposes. It has as an objective to: (1) describe the current literature on the relationship between EDS and SEP, and (2) provide recommendations for consideration of SEP in sleep medicine and biomedical research.

## 2. Materials and Methods

### 2.1. Literature Review

**Figure 1:**
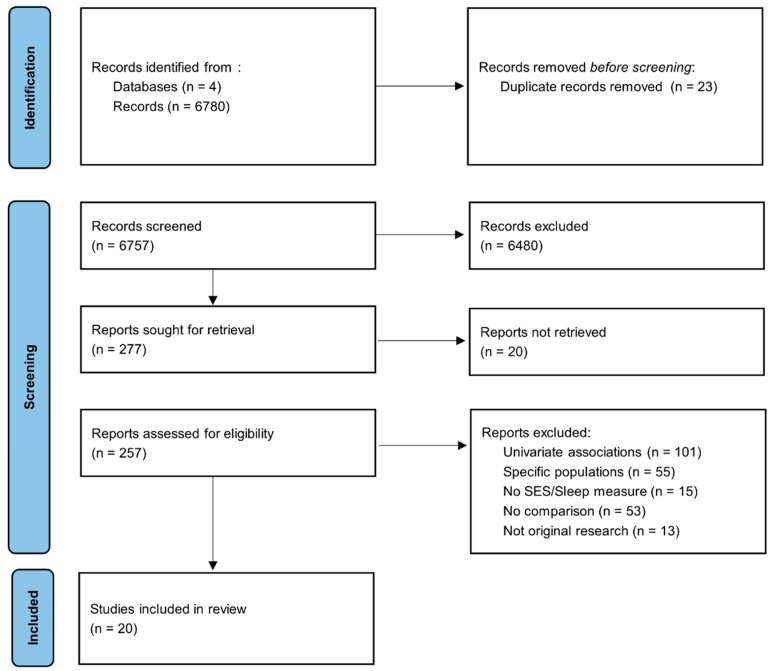
Prisma flowchart of study selection process: the relationship between EDS and SEP.

Using Prisma [32], articles included in this review were identified by screening in PubMed, Web of Science, Google scholar and Scopus. Keywords/Mesh terms used for SEP were a mix of the following: “socioeconomic”, “socioeconomic position”, “socioeconomic status”, “social position”, “social rank”, ‘’social class’’ AND the following keywords/Mesh terms used for EDS: were “sleep”, “sleep disorders”, “sleep disturbances”, “sleep complaints”, “sleep duration”, “sleepiness”, “daytime sleepiness”, “somnolence” “sleep quality”, “sleepiness”, “somnolence”, “sleep” (Figure 1). Screening ranged from January 1990 to December 2020. Only peer review articles in English with an available pdf were considered. Studies conducted before 1990 were not considered for this review, since they were mostly empirical studies conducted on animals. Clinical studies examining sleep health began after 1990. This systematic review was not pre-registered. 

### 2.2. Inclusion and Exclusion Criteria

Cross-sectional, retrospective and longitudinal studies which include individual SEP indicators such as education, income, occupation, employment status as well as perceived SES were considered, as shown in Figure 1. Full text articles were evaluated for inclusion by two independent reviewers and disagreements were resolved by discussion to reach a consensus. Proxy measures of SEP, such as composite measures and aggregate measures of SES were also included. Family SEP measures, such as parental education and household income were used when study participants were children or adolescents. Studies were excluded when they: (1) were not original research, (2) were case report/series, reviews or meta-analysis, (3) were not written in English, (4) did not have a fully accessible text or (5) included participants that already presented specific conditions at baseline (for example chronic disease, high mental health medication, pregnant women, etc.).

### 2.3. Data Extraction and Quality Rating of Studies

Eligible studies were screened to extract interrater variability measurements using Cohen’s Kappa scores to assess diagnostic agreement. Interrater variability demonstrated good agreement (κ = 0.79; 95 % CI = 0.68-0.92) as shown in Table 1. Data extracted from included studies were: Country (where research took place), year, type of population, sample size, age (mean or range depending on studies), percentage of each gender in the sample, study design, settings, SEP measures, sleep measures, findings and statistical strength of the association SEP-EDS (Odds Ratio, *p* value and r) (Table 2). SEP was considered the exposure variable and EDS the outcome variable. The National Institute of Health’s Quality Assessment Tool for Observational Cohort and Cross-Sectional Studies was used to rate the quality of included studies (Table 3). This is a tool that focuses on key factors for a critical evaluation of the internal validity of a study. SEP was considered the independent variable and sleep disturbances the dependent variable. Rating of the independent and the dependent variables of each study was performed by the two co-authors. Studies employing subjective and community SEP indicators, self-reported or non-diagnostic symptoms of EDS were downgraded. Blinding of outcome assessors was non-applicable in self-reported outcomes. Age, gender and race were considered essential confounders in statistical analyses, while body mass index or obesity were also required in studies assessing sleep-disordered breathing. Regarding overall quality rating, we calculated the proportion of positive rating on the sum of applicable criteria. Studies with <50% positive rating were judged as poor quality, ≥65% as good quality and the rest as fair quality, respectively.

## 3. Results

### 3.1. Characteristics of Studies 

Twenty studies [7,13,18,19,20,21,22,23,24,27,28,33,34,35,36,37,38,39,40] were included (Table 2), among which, nineteen were cross-sectional studies [7,13,17,18,19,20,21,22,23,27,28,32,33,34,35,36,37,38,39] and one longitudinal study [41]. Among these studies, 25.00% (n = 5) focused on children and adolescent [18,19,27,33] and the remaining 75.00% (n = 15) focused on adults and old people [7,13,18,21,22,23,24,28,34,35,36,37,38,39,40]. Among all these studies, 60.0% (n = 12) were performed in North America (ten in the USA [7,17,20,21,27,28,33,34,37] and two in Canada [19,39]), 10.00% (n = 2) in Europe (Switzerland and Norway), 10.00% (n = 2) in South America (Brazil and Peru), 10.00% (n = 2) in Oceania (Australia and New Zealand) and 10.00% (n = 2) in Asia (Japan and Iran). Ages ranged between 8 and 18 years old for children/adolescent and ranged from 18 to 102 years old for adults. Eleven studies [18,20,21,22,23,24,33,34,38,39] among these twenty articles have a majority of girls/women in their sample. Sample size in these studies varied from N = 90 ([13] until N = 33865 [37]. SEP measures presented in these studies were education [13,18,19,20,21,22,23,28,34,35,37,38,40], income [7,18,20,22,27,28,33,38], employment [7,13,17,20,22,35,38], perceived/subjective socioeconomic status (SES) [19,39], and composite index/tool using more than one SEP measure [24,36].

### 3.2. Synthesis of the Main Results

- **Education:** Lower education was related to increased perceived sleepiness [22]. Having lower education was associated with more daytime sleepiness [21]. Higher educational attainment was associated with less excessive daytime sleepiness [37]. Men with a low educational level were more likely to suffer from poor sleep quality and short sleep duration [35]. One study reported that higher level of maternal education was negatively related to EDS [20] and another one reported that EDS was not associated with educational status [40].
- **Income:** Lower income-to-needs ratio INR was related to increased sleepiness [27]. Lower INR was associated with greater daytime sleepiness [33,41]. Lower family income was associated with EDS [23]. Lower income was associated with difficulty falling asleep and having no private insurance was associated with sleep latency >30 min and non-restorative sleep [28]. The prevalence of EDS was lower in adults with a higher family income [7].
- **Employment**: Individuals employed full-time had more sleepiness than those employed part time or not employed [18]. Men with a low occupational position were more likely to suffer from poor sleep quality and short sleep duration [35]. Men with a low occupational position were also more likely to have long sleep latency [35]. Women with a low occupational position were more likely to have long sleep latency, excessive daytime sleepiness and short sleep duration [35]. One study reported that office workers had lower EDS scores than line workers [13], and another one reported that there is no association between EDS and employment status [7].
- **Perceived/subjective SES:** In children, higher subjective SES predicted less daytime sleepiness in adolescents [41], higher subjective SES was associated with fewer sleep disturbances [19]. Being in the highest deprivation quintile was a significant independent predictor of EDS [41], compared to being in the lowest deprivation quintile [24]. Lower household food security was associated with poor sleep quality, short sleep duration and excessive daytime sleepiness [36]. Subjective SES better predicted daytime sleepiness than objective SES [39]. Lower SES was also associated with more excessive daytime sleepiness [36]. One study reported that higher SES was associated with less daytime sleepiness [39] and another one reported that there was no association between SES and EDS [38].

**Table 2:** Characteristics of included studies investigating the relation between SEP and sleepiness/excessive daytime sleepiness.

Children and Adolescents < 18 Years Old										
Author	Country	Population	Sample Size	Mean or Range Age (Years)	% Women	Study Design	SES Measures	Sleep Measures	Findings	Odds Ratio, *p* Value, r
**Pallesen 2011** [20]	Norway	11th–13th grade adolescents from high schools in Hordaland county	1279	N/A	62.4	Cross-sectional	Parental education (primary school, vocational/high school, college/university)	Behaviorally induced insufficient sleep syndrome (excessive daytime sleepiness, short habitual sleep duration and sleeping considerably longer than usual during weekend/vacations)	Higher level of maternal education was negatively related to EDS	* Maternal education—EDS: OR = 0.51 * Urban living—EDS: OR= 1.52 * Grade average—EDS: *p* < 0.01
**Jarrin 2014** [19]	Canada	Children and adolescents recruited from schools and neighbourhoods in Montreal	239	8–17 (12.6 ± 1.9)	45.6	Cross-sectional	Objective SES: household income (17 categories) and highest parental education (9 categories) Subjective SES: Subjective Social Status Scale-Youth Version (two 10-rung ladders: school and society, youth reported)	Sleep quality (youth-rated, 10-point scale) Daytime sleepiness (youth-rated, Pediatric Daytime Sleepiness Scale) Sleep disturbances (parent-rated, Children’s Sleep Habits Questionnaire) Sleep duration (youth- and parent-reported)	In children, higher subjective SES predicted less daytime sleepiness in adolescents, higher subjective SES was associated with fewer sleep disturbances.	Subjective SES was associated with daytime sleepiness (*p* < 0.01)
**Bagley 2015** [27]	USA	Children recruited from semirural public schools in the southeastern US	271	11.33 ± 7.74	47	Cross-sectional	Income-to-needs ratio (computed by dividing family income by the federal poverty threshold for the same family size)	One-week actigraphy (sleep duration, night waking duration, variability in sleep schedule) School Sleep Habits Survey (Sleep/Wake Problems Scale, Sleepiness Scale)	Lower income-to-needs ratio was related to increased sleepiness.	A lower income-to-needs ratio was moderately related to increased sleepiness (*p* = 0.01)
**Tomaso 2020** [33]	USA	Adolescents members of a cohort recruited from advertisements in a small city in the Midwest US	184	11–14 (12)	50.5	Cross-sectional	Family income-to-needs ratio (dividing family income by year’s federal poverty level for a specific family size)	Sleep wake problems (Sleep–Wake Problems Behavior Scale of the Sleep Habits Survey) Daytime sleepiness (Epworth Sleepiness Scale–Revised for Children)	Lower income-to-needs ratio was associated with greater daytime sleepiness	SES significantly predicted daytime sleepiness (*p* = 0.009)
**Philbrook2020** [41]	USA	Adolescents recruited through flyers distributed at local elementary schools in semirural areas and small towns in Alabama US	252	16 (Mage = 15.79 years, SD ± 0.81), 17 (Mage = 16.79 years, SD ± 0.81), 18 (Mage = 17.73 years, SD ± 1.00) years across the three waves	53	3-wave longitudinal	Income-to-needs-ratio (INR) derived from familial income range and reported house- hold size Perceived economic well-being	Sleep–wake processes—sleep quality and daytime sleepiness (Two scales of the well-established School Sleep Habits Survey)	Family chaos is an intervening or mediator variable in longitudinal associations between indicators of lower SES and poor quality of sleep or greater daytime sleepiness	* Lower INR and poor quality of sleep (*p* < 0.01) and higher levels of chaos (*p* < 0.05) in which it acts as a mediator * Lower INR and daytime sleepiness (*p* = 0.350) where chaos is an intervening variable (*p* < 0.05) * Chaos is an intervening variable between economic well-being and both poor quality of sleep and daytime sleepiness (*p* < 0.01; *p* < 0.05)
**Adults and Old People ≥ 18 Years Old**										
**Author**	**Country**	**Sample Characteristics**	**Sample Size**	**Mean or Range Age (Years)**	**% Women**	**Type of Study**	**SES Measures**	**Sleep Measures**	**Findings**	**Odds Ratio, *p* Value, r**
**Breslau 1997** [18]	USA	Adults insured in a health maintenance organization in southeast Michigan	973	26–36	62.4	Cross-sectional	Education (<high school, high school, some college, college) Employment status (employed full-time vs. employed part time or unemployed)	Daytime sleepiness (five items from the Sleep–Wake Activity Inventory)	Those employed full-time had more sleepiness than those employed part time or not employed	N/A
**Doi 2003** [40]	Japan	Workers of a telecommunications company in Tokyo	4722	20–59	17.8	Cross-sectional	Education (< or >high school)	Excessive daytime sleepiness (ESS score >10)	EDS was not associated with educational status	* Education-EDS: OR=1.47, *p* = 0.001 * Marital status-EDS: OR = 1.61, *p* = 0.001
**Hara 2004** [23]	Brazil	1066 adults from the general population in Bambui	1066	NR	55.7	Cross-sectional	Years of education (0, 1–3, 4–7, 8+). Monthly personal income (none, <1, 1.0–1.9, ≥2.0 Brazilian minimum wages); Monthly family income (<2.0, ≥2.0 Brazilian minimum wages); Current employment situation (student, working, unemployed, retired)	Excessive daytime sleepiness three or more times per week with consequent impairment of daily activities	Lower family income was associated with EDS	* Marital status-EDS: *p* = 0.559 * Education-EDS: *p* = 0.138 * Employment status-EDS: *p* = 0.001 * Income-EDS: *p* = 0.001
**Gander 2005** [24]	New Zealand	Adults from the general population	5441	30–60	54	Cross-sectional	Area deprivation index (divided in quintiles)	EDS (Epworth Sleepiness Scale score > 10)	Being in the highest deprivation quintile was a significant independent predictor of EDS, compared to being in the lowest deprivation quintile	Ethnicity-EDS: OR = 1.55, *p* < 0.0001
**Kim 2005** [22]	USA	Adults part of a cohort of employees in five state agencies in Wisconsin	2913	35–65 (46.6 ± 7.9)	53.6	Cross-sectional	Education (some college or less, college graduate or higher)	Excessive daytime sleepiness (13-item questionnaire subjected to factor analysis)	Lower education was related to worse perceived sleepiness	Education-EDS: *p* = 0.002
**Baker 2009** [21]	USA	Women from the general US population	959	18–64 (45 ± 11.7)	100	Cross-sectional	Employment status (working full-time, working more than one job, working part time, student, homemaker, unemployed, retired or disabled) Education (high school or less, graduated from high school, some vocational or college courses, graduated from college) Household income (<$35,000, $35,000–$75,000, >$75,000)	Sleep quality (single question, dichotomous) Daytime sleepiness (single question, dichotomous)	Having lower education was associated with more daytime sleepiness	Education-EDS: *p* = 0.001
**Grandner 2013** [28]	USA	Adults from the general US population	4081	46.54 ± 16.55	48.05	Cross-sectional	Household income (above vs. below $20,000) Education level (<9th grade, 9th to 11th grade, high school graduate, some college, college graduate) Access to private health insurance (yes vs. no) Household food security (combination of 18 items and categorised as full, marginal, low, very low)	Sleep latency >30 min (yes vs. no) Insomnia symptoms (never, rarely, sometimes, often, almost always) Daytime sleepiness (never, rarely, sometimes, often, almost always) Sleep apnea symptoms (never, rarely, sometimes, frequently)	Lower income was associated with difficulty falling asleep, lower education with sleep latency > 30 min, non-restorative sleep, snorting/gasping and snoring, no private insurance with sleep latency >30 min and non-restorative sleep and lower household food security with all symptoms	* Education-EDS: OR = 1.04 * Income-EDS: OR = 1.11 *Marital status-EDS: OR = 1.24, *p* < 0.05 * Ethnicity/Race-EDS: OR = 0.8, *p* < 0.05
**Jarrin 2013** [39]	Canada	Adults recruited from advertisements in Montreal	177	30–65 (45.3 ± 6.3)	81.4	Cross-sectional	Objective SES: household income, years of education, employment status (employed vs. unemployed) Subjective SES: MacArthur Scale of Subjective Social Status (scale 1-10)	Sleep quality (PSQI Global score) Sleep latency (PSQI sleep latency subscale) Weekday sleep duration Weekend oversleep (difference between weekend and weekday total sleep duration) Daytime sleepiness (ESS)	Higher SES was associated with less daytime sleepiness. Subjective SES better predicted daytime sleepiness than objective SES.	* Household income—SES: r = 0.50, *p* < 0.01 * Years of education—SES: r = 0.26, *p* < 0.01
**Liviya NG2014** [38]	Australia	Adults recruited from 10 workplaces in Melbourne	707	40.2 ± 10.4	60	Cross-sectional	Education (nontertiary vs. tertiary) Occupation (manager, professional, associate professional, clerical or service) Income per week (≥$2000, $1600–$1999, $1000–$1599, $0–$999)	Excessive daytime sleepiness (ESS score >10)	There was no association between SES and EDS	R = 0
**Cunningham 2015** [37]	USA	Adults from the general US population		NR	NR	Cross-sectional	Educational attainment (years of completed schooling)	Sleep duration (<7, 7–8, >8 h) Fatigue (yes vs. no) Excessive daytime sleepiness (yes vs. no) Insomnia (yes vs. no)	Higher educational attainment was associated with less excessive daytime sleepiness	* Education-EDS: *p* = 0.0001
**Schwartz 2015** [36]	Peru	2682 adults from the general population of four Peruvian settings	2682	>35 (54.1 ± 18.8)	49.4	Cross-sectional	Wealth index (based on current occupation, household income, assets and household facilities)	SDB symptoms: habitual snoring (self-reported snoring at least 3 nights per week); observed apneas (pauses in breathing or choking during sleep reported by a spouse or bed partner); excessive daytime sleepiness (modified ESS score > 6)	Lower SES was associated with more excessive daytime sleepiness.	* SES-EDS: OR = 1.41, *p* = 0.006
**Stringhini 2015** [35]	Switzerland	Adults from the general population in Lausanne	3391	40–81	47.4	Cross-sectional	Educational level (high, middle, low) Occupational position (high, middle, low)	Subjective sleep assessment: sleep quality (PSQI global score > 5), sleep latency (>30 min), daytime sleepiness (ESS score > 10), sleep duration (<5 h), insomnia (from 2 items in PSQI) Objective sleep assessment: total sleep time, sleep latency, slow wave sleep, sleep efficiency, stage shifts (in-home 1-night * PSG in a subsample of 1569 participants)	Men with a low educational level or occupational position were more likely to suffer from poor sleep quality, short sleep duration and insomnia. Men with a low occupational position were also more likely to have long sleep latency. Women with a low educational level were more likely to have long sleep latency and short sleep duration. Women with a low occupational position were more likely to have long sleep latency, excessive daytime sleepiness and short sleep duration. Participants with low SES had lower sleep efficiency and higher stage shifts in PSG.	* Men with low educational and occupational position—ESS: *p* < 0.001 * Men with low occupational position have long sleep latency: OR = 4.90 * Women with low education have long sleep latency and short sleep duration: OR = 2.09; OR = 2.26 * Sleep efficiency—low SES: *p* < 0.05 * Low SES—higher stage shifts: *p* < 0.05
**Miner 2018** [34]	USA	Community-dwelling elderly persons	357	78–102 (84.2 ± 4.4)	67.8	Cross-sectional	Education (high school vs. higher)	Epworth Sleepiness Scale (≥10)	No association between education and EDS	R = 0
**Kolla 2020** [7]	USA	Community-dwelling adults	5692	18–60	27.4	Cross-sectional	Family income (low, low-average, high-average, high) Employment status (working, student, homemaker, retired)	Interview	The prevalence of EDS was lower in adults with a higher family income No association between EDS and employment status	* EDS—low income: OR: 1.21 * Low average income: OR = 1.36 * High average income: OR: 1.24 * EDS—workers, students, homemakers or retired: OR = 0.88–0.89
**Mokarami 2020** [13]	Iran	Workers in a brick factory	90	22–68 (35.6 ± 4.3)	0	Cross-sectional	Education (elementary, diploma, university) Occupational title (office vs. line workers)	Epworth Sleepiness Scale	No association between education and EDS Office workers had lower ESS scores than line workers	Occupation-EDS: *p* = 0.0001

* SES = socioeconomic status; PSQI = Pittsburgh Sleep Quality Index; DSM = Diagnostic and Statistical Manual of Mental Disorders; SDB = sleep-disordered breathing; ESS = Epworth Sleepiness Scale; EDS = excessive daytime sleepiness; OSA = obstructive sleep apnea; ICD = International Statistical Classification of Diseases and Related Health Problems; BIISS = behaviorally induced insufficient sleep syndrome, NR = Non Reported, PSG = polysomnography.

**Table 3:** Quality rating of included studies using the National Institute of Health’s Quality Assessment Tool for Observational Cohort and Cross-Sectional Studies.

Study	Q1	Q2	Q3	Q4	Q5	Q6	Q7	Q8	Q9	Q10	Q11	Q12	Q13	Q14	Quality Rating
**Breslau 1997** [18]	Y	Y	Y	Y	N	N	N	Y	Y	N	N	NA	NA	Y	Fair
**Doi 2003** [40]	Y	Y	Y	Y	N	N	N	N	Y	N	N	NA	NA	Y	Fair
**Hara 2004** [23]	Y	Y	Y	Y	N	N	N	Y	Y	N	N	NA	NA	N	Fair
**Gander 2005** [24]	Y	Y	Y	Y	N	N	N	Y	N	N	N	NA	NA	Y	Fair
**Kim 2005** [22]	Y	Y	NR	Y	N	N	N	N	Y	N	N	NA	NA	Y	Poor
**Baker 2009** [21]	Y	Y	N	Y	N	N	N	Y	Y	N	N	NA	NA	Y	Fair
**Pallesen 2011** [20]	Y	N	Y	Y	N	N	N	Y	Y	N	Y	NA	NA	Y	Fair
**Ansarin 2013** [42]	Y	Y	Y	Y	N	N	N	Y	Y	N	N	NA	NA	Y	Fair
**Grandner 2013** [28]	Y	Y	Y	Y	N	N	N	Y	Y	N	N	NA	NA	Y	Fair
**Jarrin 2013** [39]	Y	N	NA	Y	N	N	N	Y	Y	N	N	NA	NA	Y	Poor
**Jarrin 2014** [19]	Y	N	NA	Y	N	N	N	Y	Y	N	N	NA	NA	Y	Poor
**Liviya NG 2014** [38]	Y	Y	NR	Y	N	N	N	Y	Y	N	N	NA	NA	Y	Fair
**Bagley 2015** [27]	Y	Y	Y	Y	N	N	N	Y	Y	N	Y	Y	NA	Y	Good
**Cunningham 2015** [37]	Y	Y	Y	Y	N	N	N	Y	Y	N	N	NA	NA	Y	Fair
**Schwartz 2015** [36]	Y	Y	N	Y	N	N	N	Y	Y	N	N	NA	NA	Y	Fair
**Stringhini 2015** [35]	Y	Y	Y	Y	N	N	N	Y	Y	N	Y	NR	NA	Y	Fair
**Miner 2018** [34]	Y	Y	Y	Y	N	N	N	N	Y	N	N	NA	NA	Y	Fair
**Tomaso 2018** [33]	Y	N	Y	Y	N	N	N	N	Y	N	N	NA	NA	Y	Poor
**Kolla 2020** [7]	Y	Y	Y	Y	N	N	N	Y	Y	N	Y	Y	NA	Y	Good
**Mokarami 2020** [13]	Y	Y	Y	Y	N	N	N	Y	Y	N	Y	Y	NA	Y	Good
**Philbrook 2020** [41]	Y	Y	Y	Y	N	N	N	Y	Y	N	Y	Y	NA	Y	Good

Y = Yes; N = No; CD = cannot determine; NA = not applicable; NR = not reported.

## 4. Discussion

The aim of the present systematic review of observational cohort longitudinal and cross-sectional studies is to provide the current status of knowledge on the link between socioeconomic position (SEP) and excessive daytime sleepiness (EDS). Overall, a lower educational level, a lower income and full-time employment were associated with more excessive daytime sleepiness (Table 2). EDS symptoms are prevalent in women, especially those with a low income or no job [21]; and finally, children and adolescents with difficult living conditions or working part time reported more sleep disturbances [19,23,27].

- Association between EDS and SEP in children and minors

EDS was evaluated among young children and minors, a population in which development and growth rely on many physiological processes, including sleep. However, their sleep needs are often unmet. The actual average number of hours slept for minors was 6.7 h on weekdays and 7.4 on weekends [17,22,38,42]. EDS was inversely related to the number of hours of sleep and was associated with lower grade average and elevated scores of anxiety and depression [13,17,43]. These results, along with other studies, show that sleep disturbances are linked to poor academic performance, cognitive impairment and mental issues [37,43]. Disruptive sleep environment, conditions non-conducive to sleep or great worries could explain why children living in disadvantageous conditions have more sleep disturbances [21]. Moreover, the omnipresence of technological devices, the commotion of urban environment as well remunerated work influences their sleep duration and quality [13,43].

- Association between EDS and SEP in adults

Low SEP was strongly associated with poor sleep quality in adults. Important factors such as workload, varying shifts, family commitments or financial stress could account for the observed disparity in the burden of sleep disturbances on people with low SEP [35]. This association persists even after the adjustment for other sociodemographic, psychological or behavioral factors [35]. 

Moreover, EDS symptoms seem to be more frequent in women, especially in those with a lower family income or no job [21]. Women consider psychological factors to be the most important causes of sleep disturbances [20,44,45], therefore a low SEP could be perceived as a bigger burden by females rather than males. Furthermore, anxiety is significantly more common in females. However, psychological stress could be a consequence rather than a cause [44,46]. Women included in sleep health studies are often of the childbearing age, therefore, the higher number of sleep disturbance complaints could be due to the presence of young children [27,44,45].

- Association between EDS and SEP in people with mental, physical and functional disability

Excessive daytime sleepiness was also significantly associated with mental disorders, chronic physical conditions and greater functional disability among vulnerable populations [7]. Sleep disturbance is commonly observed in a wide range of psychiatric disorders reported by disadvantaged communities [17,45] and sleep disturbances increase negative emotions and decrease positive emotional responses among these communities more than their pairs [47]. 

EDS also seems to be more prevalent in low SEP individuals with chronic conditions than high SEP people with the same chronic conditions [48]. These chronic conditions such as diabetes, arthritis and obesity have been linked to poor quality of sleep [7,36,49], which in turn partly explains the high prevalence of sleep disturbances that have been observed in the elderly population [22,38,46].

- Association between EDS and SEP in the elderly

The lack of a significant association between sleep disturbances and SEP in geriatric research may be due to the presence of comorbidities. Multiple chronic diseases can cause bodily pain and can cause sleep disturbance independently of SEP. Reports of daily bodily pain account for 35% of complaints of sleep disturbance, including EDS [48]. 

Other factors that contribute to sleep problems in old age independently of SEP are changes in social engagement and lifestyle [49]. As their daily demands diminish and lifestyle becomes more sedentary, alteration in their sleep might not be as noticeable [50]. Furthermore, there are normal physiological alterations in sleep that arise with older age [51]. These changes include shortened nocturnal sleep duration as well as a higher frequency of daytime napping [51]. Although more studies are required to understand the association between sleep disturbances and SEP, the findings of this review point out a potential relationship that deserves more investigations. As previously mentioned, the older population often suffers from multiple diseases, therefore, those with no health insurance might be more worried about their medical expenses, which in turn might impair their sleep quality [51].

- Recommendation for future sleep research

**Figure 2:**
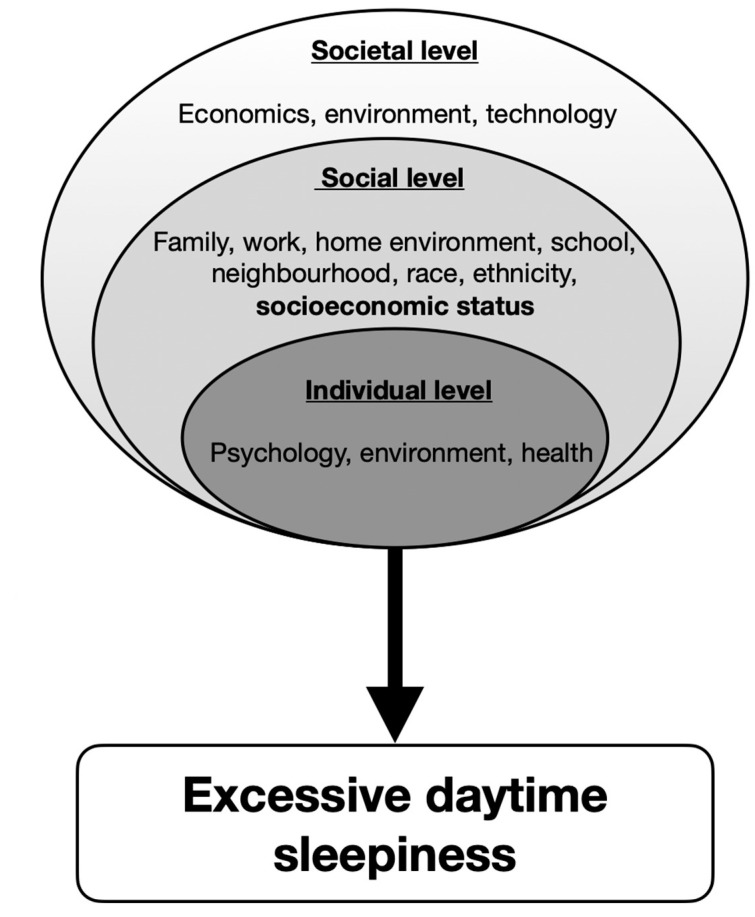
The biopsychosocial model of excessive daytime sleepiness.

Given the wide range of negative impacts associated with EDS, further research needs to be conducted to raise awareness among healthcare workers and improve clinical guidelines. Social class is a strong predictor of health outcomes and yet, it is a variable that remains particularly underexamined in sleep science. It is vital to gather more information about the contribution of other SEP parameters on EDS, such as access to insurance, ethnicity or immigration status. The temporality of the relationship between EDS and SEP needs to be further assessed in longitudinal studies, rather than cross-sectional, which can discern the direction of the interaction and allow clear causality interpretations on how SEP influences EDS. In order to implement and design clinical and public health initiatives to improve the overall population health and reduce the gap in health disparities, inclusion of objective measures of sleep, obtained by actigraphy or polysomnography should be included in future research [52,53]. Considering that sleep is an intricate and individual process that is heavily influenced by complex and dynamic interrelations across a lifetime, the development of a multivariable model that allows to consider and describe the various modifiable factors that take play in EDS would lead to better novel clinical interventions. Figure 2 presents a biopsychosocial model of EDS that considers the various interactions between societal, social and individual factors. 

The recent shift in the societal paradigm accompanied by technological advances render our lifestyles incompatible with good sleep. The incessant need for productivity and the increasing hours of work have impacted the quality and quantity of sleep that affects mostly developed countries. Furthermore, the abundant availability of technological devices has also diminished the duration of sleep that affects people of all ages. The proliferation of screens and electronics increases our exposure to blue wavelengths, which in turn suppresses the secretion of melatonin. This phenomenon has been linked to diabetes, obesity, as well as depression. 

On a smaller scale, SEP reflects the conditions of an individual’s environment, such as their community, workspace and family. Sleep is an intricate process that is heavily affected by environmental cues; thus, a lower SEP is linked to more sleep disturbances, more worries and anxiety that all lead to poor sleep hygiene. Socioeconomic disparity creates a greater gap between different individuals, based on their age, sex or ethnicity. 

Although sleep is impacted by the larger spheres of society and community, it is a personal physiological function. Therefore, the lifestyle, behavior and psychology of the individual is of the utmost importance. Behavioral risk factors such as smoking, drinking, drug use or eating disorders are linked with a lower SEP as well as with poor health outcomes (including sleep health). Disadvantageous living conditions impact sleep health directly and indirectly via mental health. The bidirectional association between sleep and mental health is significant and could account for a large part of sleep disorders. 

## 5. Conclusions

Modern medical care revolves mainly on a biomedical model where quantitative measurements are very often considered the gold standard for clinical practice as well as public health administrators. However, this approach has limitations in assessing long-term health outcomes and their relationship with socioeconomic disparities. SEP is already considered as an important determinant for many health outcomes, but if SEP is embedded in experimental design in psychosomatic research, biomedical research and clinical practice as a constant variable regardless of outcome, it will move forward future investigations. It is a challenge to capture the multidimensional nature of SEP, which highlights the need for better measures that would be comparable among various populations and across different types of studies. Future research is still required to understand how socioeconomic status, social class and socioeconomic position influence sleep health while analyzing possible mediators and moderators. Achieving a sustainable and wide impact on sleep disparities is a challenging feat that requires a coordination of efforts of various systems to instill a perceivable change. 

## Figures and Tables

**Table 1:** The data of kappa agreement during the selection and extraction of data from the studies.

Articles	Included	Kappa		Articles	Included	Kappa		Articles	Included	Kappa		Articles	Included	Kappa
1	Y	0.82		36	N	0.82		71	N	0.76		106	N	0.72
2	N	0.72		37	N	0.72		72	N	0.72		107	Y	0.76
3	N	0.9		38	N	0.72		73	N	0.73		108	N	0.74
4	N	0.77		39	N	0.79		74	N	0.80		109	N	0.78
5	N	0.80		40	N	0.81		75	N	0.86		110	N	0.83
6	N	0.74		41	N	0.65		76	N	0.74		111	N	0.82
7	N	0.84		42	N	0.89		77	N	0.69		112	N	0.81
8	N	0.67		43	N	0.84		78	N	0.72		113	N	0.78
9	N	0.80		44	N	0.78		79	N	0.74		114	N	0.74
10	N	0.67		45	N	0.83		80	N	0.81		115	N	0.76
11	N	0.85		46	N	0.68		81	N	0.76		116	N	0.83
12	N	0.79		47	N	0.67		82	N	0.74		117	N	0.74
13	N	0.78		48	N	0.76		83	N	0.72		118	N	0.68
14	Y	0.86		49	N	0.85		84	N	0.74		119	N	0.62
15	N	0.92		50	N	0.84		85	N	0.73		120	N	0.68
16	N	0.68		51	N	0.80		86	N	0.74		121	N	0.72
17	N	0.81		52	N	0.70		87	N	0.75		122	Y	0.76
18	N	0.81		53	Y	0.92		88	N	0.76		123	N	0.74
19	N	0.64		54	Y	0.84		89	N	0.82		124	N	0.82
20	N	0.69		55	N	0.83		90	N	0.63		125	N	0.74
21	N	0.82		56	N	0.78		91	N	0.68		126	N	0.71
22	N	0.81		57	N	0.71		92	N	0.71		127	N	0.62
23	N	0.76		58	N	0.82		93	N	0.74		128	N	0.78
24	N	0.74		59	N	0.79		94	N	0.75		129	N	0.81
25	N	0.79		60	N	0.71		95	N	0.82		130	N	0.72
26	N	0.75		61	N	0.84		96	N	0.68		131	N	0.73
27	N	0.81		62	N	0.86		97	N	0.74		132	N	0.76
28	N	0.86		63	N	0.90		98	N	0.76		133	Y	0.85
29	N	0.90		64	N	0.76		99	N	0.82		134	N	0.74
30	N	0.68		65	N	0.78		100	N	0.86		135	N	0.67
31	N	0.75		66	Y	0.74		101	N	0.81		136	N	0.64
32	N	0.88		67	N	0.90		102	Y	0.78		137	N	0.72
33	N	0.65		68	N	0.75		103	N	0.74		138	Y	0.74
34	N	0.78		69	N	0.76		104	N	0.76		139	N	0.76
35	N	0.74		70	N	0.81		105	N	0.82		140	N	0.74
141	N	0.71		176	N	0.74		194	N	0.76		229	N	0.75
142	N	0.87		177	N	0.74		195	Y	0.78		230	N	0.81
143	N	0.74		178	N	0.82		196	N	0.74		231	Y	0.86
144	N	0.64		179	Y	0.74		197	N	0.90		232	N	0.76
145	N	0.74		180	N	0.71		198	N	0.75		233	N	0.74
146	N	0.69		181	N	0.62		199	N	0.76		234	N	0.71
147	N	0.72		182	N	0.78		200	N	0.81		235	N	0.87
148	N	0.71		183	N	0.81		201	N	0.76		236	N	0.74
149	N	0.74		184	N	0.72		202	N	0.72		237	N	0.64
150	N	0.67		185	N	0.73		203	N	0.73		238	N	0.74
151	N	0.82		186	N	0.76		204	N	0.80		239	N	0.69
152	N	0.67		187	N	0.71		205	N	0.86		240	N	0.72
153	N	0.64		188	N	0.82		206	N	0.90		241	N	0.71
154	Y	0.90		189	N	0.79		207	N	0.77		242	N	0.74
155	Y	0.76		190	N	0.71		208	N	0.80		243	N	0.74
156	Y	0.82		191	N	0.84		209	N	0.74		244	N	0.82
157	N	0.74		192	N	0.86		210	N	0.84		245	N	0.74
158	N	0.76		176	N	0.74		211	N	0.67		246	N	0.71
159	N	0.68		177	N	0.74		212	N	0.80		247	N	0.62
160	N	0.85		178	N	0.82		213	N	0.67		248	N	0.78
161	N	0.74		179	Y	0.74		214	N	0.85		249	Y	0.81
162	N	0.67		180	N	0.71		215	N	0.79		250	N	0.62
163	N	0.64		181	N	0.62		216	N	0.78		229	N	0.75
164	N	0.72		182	N	0.78		217	N	0.86		230	N	0.81
165	N	0.74		183	N	0.81		218	N	0.92		231	Y	0.86
166	N	0.76		184	N	0.72		219	Y	0.68		232	N	0.76
167	N	0.74		185	N	0.73		220	N	0.81		233	N	0.74
168	N	0.71		186	N	0.76		221	N	0.81		234	N	0.71
169	N	0.87		187	N	0.71		222	N	0.64		235	N	0.87
170	N	0.74		188	N	0.82		223	N	0.69		236	N	0.74
171	N	0.64		189	N	0.79		224	N	0.82		237	N	0.64
172	Y	0.74		190	N	0.71		225	N	0.81		238	N	0.74
173	N	0.69		191	N	0.84		226	N	0.76		239	N	0.69
174	N	0.72		192	N	0.86		227	N	0.74		240	N	0.72
175	Y	0.71		193	N	0.90		228	N	0.79		241	N	0.71
242	N	0.74		246	N	0.71		250	N	0.62		254	N	0.68
243	N	0.74		247	N	0.62		251	N	0.75		255	N	0.74
244	N	0.82		248	N	0.78		252	N	0.68		256	N	0.76
245	N	0.74		249	Y	0.81		253	N	0.75		257	N	0.78

Y = Yes; N = No.
